# Special considerations in conducting clinical trials of chronic pain management interventions in children and adolescents and their families

**DOI:** 10.1097/PR9.0000000000000649

**Published:** 2018-04-10

**Authors:** Tonya M. Palermo, Susmita Kashikar-Zuck, Stefan J. Friedrichsdorf, Scott W. Powers

**Affiliations:** a Center for Child Health, Behavior, and Development, Seattle Children's Research Institute, Seattle, WA, USA; b Department of Anesthesiology and Pain Medicine, University of Washington, Seattle, WA, USA; c Division of Behavioral Medicine and Clinical Psychology, Cincinnati Children's Hospital Medical Center, Cincinnati, OH, USA; d Department of Pediatrics, University of Cincinnati College of Medicine, Cincinnati, OH, USA; e Department of Pain Medicine, Palliative Care and Integrative Medicine, Children's Hospitals and Clinics of Minnesota, Minneapolis, MN, USA

**Keywords:** Chronic pain, Clinical trials, Children and adolescents

## Abstract

**Introduction:**

Disabling chronic pain is a common experience for children and adolescents. However, the evidence base for chronic pain interventions for youth is extremely limited, which has hindered the development of evidence-based practice guidelines for most pediatric chronic pain conditions.

**Objectives:**

To review and provide recommendations on clinical trial design and evaluation in children and adolescents with chronic pain.

**Methods:**

In this article, we summarize key issues and provide recommendations for addressing them in clinical trials of chronic pain interventions in children and adolescents and their families.

**Results:**

To stimulate high-quality trials of pediatric chronic pain management interventions, attention to key issues including sample characterization, trial design and treatment administration, outcome measurement, and the ethics of intervening with children and adolescents, as opposed to adults with chronic pain, is needed.

**Conclusion:**

Future research to develop interventions to reduce or prevent childhood chronic pain is an important priority area, and requires special considerations in implementation and evaluation in clinical trials.

## 1. Introduction

### 1.1. Prevalence and impact of pediatric chronic pain

Pain that persists for longer than 3 months in children and adolescents is common; epidemiological studies estimate that 5% to 8% of children have severe and disabling chronic pain.^40^ The functional consequences of chronic pain on children and adolescents are reflected in missed school days, limited social and athletic activities, and emotional distress.^59^ Indeed, the costs of chronic pain include not only reduced quality of life for children but also lost work productivity and high costs to parents and caregivers. The total direct cost of moderate–severe pediatric chronic pain in the United States is extrapolated to $19.5 billion per year.^33^ Because longitudinal data demonstrate that childhood chronic pain places individuals at significant risk of developing or continuing with chronic pain, physical symptoms, and psychiatric complaints into adulthood,^86^ effective treatment of pain in childhood is critical for preventing or lessening the enormous societal impact of adult chronic pain.

Chronic pain includes persistent (ongoing) and recurrent (episodic) pain in children with underlying health conditions (eg, inflammatory bowel disease, sickle cell disease [SCD], juvenile idiopathic arthritis) and primary pain disorders (eg, primary headaches, centrally mediated abdominal pain syndrome, widespread musculoskeletal pain) as well as complex regional pain syndrome.^29,79^ A significant number of children experience both entities, ie, “acute-on-chronic” pain such as children with SCD who may have ongoing daily widespread pain superimposed with acute vaso-occlusive pain exacerbations. Clinical studies have also demonstrated altered pain pathways in the central nervous system associated with overall heightened pain sensitivity (thought to be a central sensitization phenomenon) in patients with many types of localized and diffuse chronic pain syndromes such as chronic low back pain, neck pain, headaches, widespread musculoskeletal pain, and irritable bowel syndrome.^29,88^

### 1.2. Chronic pain interventions in children and adolescents

Our goal in this review is to provide a comprehensive overview of special considerations in designing and conducting clinical trials for chronic pain interventions in children and adolescents. Chronic pain interventions for children and adolescents encompass a variety of single and multi-modal treatments including pharmacological (eg, analgesics, antidepressants, anticonvulsants, antimigraine medications), psychological (eg, cognitive-behavioral therapy), physical therapy (eg, aerobic exercise, strengthening, graded motor imagery), and complementary and integrative health interventions (eg, biofeedback, self-hypnosis, yoga). Treatments have been delivered in different settings including the home,^32,39,66,76^ in schools,^25,52^ in outpatient clinics,^48,73,87^ in hospitals,^31^ and in inpatient intensive rehabilitation settings.^20,38,55,56,64^ Although many of these interventions are routinely used in clinical care, most have not been tested in rigorous trials in pediatric populations.

Despite the high prevalence, cost, and impact of chronic pain in the general population, the evidence base for chronic pain interventions is very limited. This is particularly pronounced for clinical trials of pediatric chronic pain interventions. Children have historically been underrepresented in all clinical trials compared with adults. Adult publications of randomized controlled trials (RCTs) are increasing at a faster rate than pediatric RCTs (90.5 RCTs per year vs 16.9 RCTs per year) in almost all specialties.^11^ For example, in one analysis among registered clinical drug trials, although 59.9% of the disease burden for 9 selected conditions was attributable to children, only 12.0% of trials were pediatric.^8^ Fewer pediatric trials are funded by industry and thus, most investigations rely on funding from government and nonprofit organizations.^8^ As another example of the limited pediatric chronic pain intervention research, the Cochrane Pain, Palliative and Supportive Care Review Group contains only 13 titles devoted to chronic noncancer pain in children compared with over 150 titles in adults. Lack of data on treatment efficacy has hindered the development of evidence-based treatment guidelines for most pediatric chronic pain conditions.

The lack of high-quality trials testing the full range of treatment options specifically for children and adolescents with chronic pain is a major concern. Indeed, the United States Federal Pain Research Strategy (https://iprcc.nih.gov/sites/default/files/FPRS_Research_Recommendations_Final_508C.pdf) has specifically prioritized the understanding of mechanisms of childhood chronic pain and effective chronic pain management in children. Children are not simply “little adults” and application of pharmacologic and nonpharmacologic treatments that are based on evidence from adult studies can often be inappropriate. For example, differences in pharmacodynamics, pharmacokinetics, and pharmacogenomics in a developing nervous system may result in suboptimal effects, adverse drug responses, and toxicity. Despite recognition of the need to conduct trials of medicines used in children and legislation to facilitate this, there continue to be a dearth of pediatric trials.^8^ Particularly concerning, Bourgeois et al.^8^ found a relative paucity of registered pharmacokinetic/dynamic and safety assessments in clinical trials conducted in children compared with adults. Clearly, more attention to this area is needed. In addition to biologic differences, psychosocial and developmental factors in pediatric populations are unique and must be considered in designing nonpharmacologic intervention studies.^85^ Although there are a larger number of trials of integrative (nonpharmacologic), primarily psychological interventions for pediatric chronic pain, the quality of these trials for the most part has been low due to numerous methodological limitations.

Fortunately, there has been a growing interest in stimulating high-quality trials of pediatric pain management interventions and recognition that differences exist in sample characterization, trial design and treatment administration, outcome measurement, and in the ethics of clinical trials with children and adolescents, as opposed to adults with chronic pain. Acute pain interventions for children have been considered separately and other groups have defined unique considerations in conducting acute pain trials in children.^7^ However, less attention has been devoted to the special considerations in clinical trials of pediatric chronic pain interventions with the exception of recommendations on selection of core outcome domains and measures for clinical trials of pediatric chronic pain management interventions (Ped-IMMPACT).^57^ Furthermore, some basic considerations in clinical trial methodology for children and adolescents with headache have been published.^2^ In this article, we discuss special considerations and provide recommendations concerning the conduct of clinical trials for chronic pain interventions in children and adolescents and their families.

## 2. Sample characterization

Important differences between children and adults exist in the signs, symptoms, and diagnosis of chronic pain conditions as well as the availability of samples. Some chronic pain conditions that are well-characterized in adulthood such as temporomandibular joint disorder and chronic low back pain are not as prevalent in childhood^29,50^ and others (such as widespread musculoskeletal pain/juvenile fibromyalgia) may not have the identical clinical signs, symptoms, and comorbidities as in adults.^45^ An issue that arises in pediatric trials is the clarity and precision with which pediatric pain diagnoses/subtypes are defined in a research study. In contrast to most trials in adult chronic pain, which typically focus on a single pain condition (eg, chronic low back pain, fibromyalgia, migraine), treatment studies in children vary greatly in terms of whether they include a heterogeneous sample of pediatric pain conditions (including headache disorders, abdominal pain, regional or widespread musculoskeletal pain) or more narrowly defined single pain conditions. This is due to the combined issues of the lack of available consensus definitions of pediatric chronic pain disorders, the limited number of treatment centers, paucity of funding for pediatric trials, and in some instances, smaller available samples. Consensus definitions of chronic pain conditions exist for a few conditions that tend to have a high prevalence in pediatric populations such as functional abdominal pain^49^ and primary headaches (tension headaches and migraines)^37^ and correspondingly, there are more single condition trials within these conditions. Overlapping pain conditions (ie, presence of more than one chronic pain condition such as migraine and irritable bowel syndrome) are also of importance in both pediatric and adult pain research and care, but consensus is lacking on classification. There is a need for more clarity in classification of pediatric pain conditions to increase understanding of treatment response in well-defined patient groups. Ideally, this would also allow for studying longitudinal trajectories of pediatric with related adult syndromes to understand whether pain interventions are effective across the lifespan. The ability to connect pediatric to adult pain conditions through classification is particularly important during the developmental transition from older adolescence to young adulthood, when care shifts to adult pain care providers.^75^

There are clearly advantages and disadvantages to each of these diagnostic approaches. Heterogeneous samples allow for greater ease of recruitment and generalizability of findings, whereas focusing on specific pain subtypes allows for tailoring of treatment for the specific condition and examination of potential mechanistic factors and response in well-specified subgroups. The lack of consistency in diagnostic terminology complicates the issues at hand further. The field needs more clarity in terms of classification of pediatric chronic pain conditions because this equally impacts the design and interpretation of trials of pharmacological and integrative (nonpharmacological) interventions, singly or in combination.

### 2.1. Pain classification

Progress has been made in pain classification that may improve efforts to characterize pediatric pain samples such as through the recent publication of an evidence-based chronic pain classification system, the ACTTION-APS Pain Taxonomy (AAPT).^28^ The resultant pain taxonomy framework developed through this initiative incorporates knowledge of biopsychosocial mechanisms and classifies chronic pain conditions along 5 dimensions including (1) core diagnostic criteria, (2) common features, (3) common medical and psychiatric comorbidities, (4) neurobiological, psychological, and functional consequences, and (5) putative neurobiological and psychosocial mechanisms, risk factors, and protective factors. This framework, comprehensively described in the study by Fillingim et al.,^28^ is intended for use across the lifespan with specific developmental issues and differences in signs and symptoms noted for each pain condition. As an example, Dampier et al.^16^ used the AAPT criteria for classifying chronic pain associated with SCD. These criteria could enhance clinical trials for pediatric SCD chronic pain by providing standardized inclusion criteria, which has been a major limiting factor in studies of pain management interventions in children and adolescents with SCD. Further work is needed to translate the AAPT classification system into a clinically useful application in the medical setting.

### 2.2. Recruitment considerations

Recruitment for clinical trials in pediatric pain has included various strategies from the hospital, community, primary care practices, and from tertiary care/subspecialty clinics. An advantage to recruiting from the community and primary care practices is access to patients with a range of pain and symptom severity that can make results of trials more generalizable. However, one challenge in recruiting from primary care services is that recognition and treatment of pediatric chronic pain is often not a part of pediatric primary care medical training^84^; hence, pediatricians are often hesitant to diagnose primary pain conditions without consulting with subspecialists to rule out other diagnoses that may be producing the pain. For example, if a child complains of recurrent headaches or abdominal pain, they are often referred to a neurologist or gastroenterologist to determine if the pain is secondary to another medical condition or disease. Recruitment from subspecialty clinics has the advantage of clearer classification/diagnosis of the pain syndrome. One potential disadvantage is that patients recruited from subspecialty/tertiary care may over-represent those with more severe or resistant symptoms or with comorbid psychiatric conditions. This could have implications for how well they respond to treatment and also make results of a trial less generalizable to those with less severe symptoms. However, choice of inclusion and exclusion criteria, as well as a focus on referrals that are directly made by primary care providers (as opposed to other specialists or second opinions) can attenuate these concerns.^70,71^

## 3. Trial design and treatment delivery

Several issues should be considered in trial design and treatment delivery in pediatric chronic pain such as inclusion of parents/caregivers, sample size, recruitment and retention challenges, novel treatment delivery settings including remote treatment delivery using digital health interventions and delivery of treatment in the school setting, and consideration of adequate control groups and placebo effects.

### 3.1. Inclusion of parents/caregivers

A unique aspect of clinical trials for chronic pain interventions in children is that the decision to participate in the trial as well as the receipt of treatments (eg, taking a medication or implementing a behavioral treatment) involve the parents/caregivers. We discuss specific issues pertaining to ethical requirements of parent involvement in the section on ethical issues. Because parents influence their child's adjustment to chronic pain, they are a unique and integral part of pediatric chronic pain treatment.^65^ Parents also serve a critical caregiving role in administration of treatments and making decisions about health care. For medication and nonmedication trials, issues related to treatment adherence are important considerations. In particular, adolescence is a vulnerable time for nonoptimal adherence to treatment regimens,^72^ and concerted efforts may be needed to obtain maximal compliance, with parental supports often included. For example, a pediatric clinical trial may require participation of at least one parent who commits to attending treatment visits or who monitors medication intake as a measure of compliance in a medication trial.

In comparison with adult trials, where treatments focus primarily on the patient, an advantage (but also a complexity) to pediatric trials is the caregiver's participation in treatment. Parent involvement can help promote engagement and facilitate a more supportive and adaptive home environment for more rapid uptake of treatment recommendations, and is recommended whenever feasible. For example, in a cross-over trial of cognitive-behavioral therapy and self-monitoring in youth with widespread musculoskeletal pain, Kashikar-Zuck et al.^47^ included parents in several treatment visits over the 16-week trial and had an excellent retention rate (90%), which they attributed in part to the involvement of parents. It is quite rare for adult intervention studies to be able to access the home and work environment for chronic pain treatment, which may in some ways limit the uptake or impact of treatment in daily life.

Moreover, there is considerable interest in the psychological treatment literature in the development of parent interventions for youth with chronic pain to address family communication, parent distress, and to promote change in parenting behaviors.^62^ In this context, the parent is seen as the agent of change and the intervention is designed to modify parent emotions, behaviors, or cognitions directly through interventions such as problem-solving therapy and cognitive-behavioral therapy interventions. Regarding trial design for these types of interventions, the parent may be the primary participant and the child may or may not receive concomitant treatment in the study. Important considerations include the choice of outcome measures to reflect parent and family outcomes targeted by the intervention as well as the intended downstream effects of parent intervention on the child's pain and mental health outcomes.

### 3.2. Sample size

Despite the improving quality of trials in pediatric chronic pain in recent years, Cochrane reviews have noted the continuing methodological shortcomings due to small sample sizes.^21,41,46,60^ In fact, there is a paucity of trials with adequate power conducted in pediatric chronic pain from which more decisive conclusions can be made. This is true for pediatric clinical trials in all conditions, where sample sizes typically include fewer than 100 participants.^44^ Planning a clinical trial in pediatric chronic pain raises some special concerns regarding sample size that need careful consideration. First, pediatric chronic pain is not as widely prevalent as chronic pain in adults, and there are far fewer treatment centers. Therefore, obtaining large samples of patients in a single geographic area is challenging. Hence, multisite studies are becoming increasingly common as the field advances and greater rigor is expected. This clearly raises the cost and complexity of a pediatric pain clinical trial. Multisite studies require personnel to be hired and trained in standardized procedures for assessment and treatment delivery across sites, blinding and quality control procedures often require additional staffing, centralized databases and proper database management requires extensive investments of time and funds, regulatory guidelines at different institutions can vary, and the increased emphasis on using centralized/single institutional review board or ethics committees for clinical trials (a requirement in United States National Institute of Health funded trials) can add its own complexities. However, this investment of resources is likely to pay rich dividends in terms of rigorous and definitive trials that provide clear direction for effective treatment of chronic pain in childhood—potentially altering trajectories of chronic pain and disability into adulthood and ultimately reducing the burden of chronic pain through the lifespan.

Two additional solutions to address the sample size challenges include (1) the formation of networks and registries to provide the infrastructure needed to conduct larger trials and to pool data, and (2) the use of novel designs and statistical methods to minimize sample sizes needed. International pediatric trial networks have been established in many countries. For example, in Australia, all pediatric (and adult) pain clinics are required to participate in the Electronic Persistent Pain Outcomes Collaboration (ePPOC), which involves data collection using a standard set of data items and assessment tools. In North America, the Childhood Arthritis and Rheumatology Alliance (CARRA) has 70 registry sites and is being used for patient recruitment into multicenter trials of treatments for juvenile arthritis and other pediatric rheumatic diseases. There are also several design and statistical methods that help to reduce necessary sample sizes. In pediatric pharmacokinetic studies, there are several examples of innovative trial design techniques for reducing the number and volume of samples required^44^ as well as using approaches to collect pharmacokinetic samples from children receiving treatment as part of their routine clinical care. Moreover, the use of appropriate statistical methods such as linear mixed-effects modeling to efficiently accommodate missing data can serve to maximize the value of the information obtained.

### 3.3. Recruitment and retention challenges

Participant burden is an important concern in pediatric research. Children may not have the motivation, cognitive capacity, or availability to participate in time-intensive protocols. Also, the demands of school and after-school activities, reliance on parents or other family members for transportation, and compliance in completing assessment and treatment protocols (eg, difficulty swallowing tablets) are all issues that must be considered in designing feasible trials for children with chronic pain. Parents may also be reluctant to enroll their child in a trial with unknown benefits and concerns about side effects (eg, black box warnings for antidepressant medication use in children). Moreover, there are specific pediatric pain populations for which obtaining adequate recruitment and participation in clinical trials is particularly challenging, such as in youth with SCD. In studies in the pediatric sickle cell population, who are mostly African American in the United States, low rates of recruitment (eg, as few as 13% of available samples) have been reported.^5,78,80^ Indeed, a key barrier identified to participation in intervention studies by patients with SCD is reaching them by phone and scheduling study visits. There are also significant challenges of enrolling African American youth and their parents into intervention research where broader socioeconomic barriers, including mistrust and misunderstanding of research,^81^ lack of perceived benefits from research participation, and potentially stressful home environments, may contribute to low enrollment.^17^

In all pediatric populations, attitudes about participation in trials are important to consider. There has been general reluctance about involving children in trials because of fears of harming children by exposing them to uncertain treatment effects.^9,18,19^ A number of strategies may help address concerns about participation in clinical trials including incorporating user-centered design and community-based participatory research approaches that involve patients and other stakeholders in the research process at all levels. This approach can be particularly valuable for addressing disparities in research participation. An example of community-based participatory research in pediatric perioperative care with Latino youth undergoing surgery and their families is described by Rosales et al.^74^ with the goal to reduce disparities in perioperative intervention approaches in minority populations.

To specifically address issues with participant retention, successful strategies used in pediatric clinical trials have included asking for multiple forms of contact information,^10^ providing escalating incentives for completion of multiple follow-up assessments, and using varied patient contact strategies to remind participants to complete study visits including phone, short message service text message, social media, and email.^53^ It is also essential for strategies to be used to obtain posttreatment measures on all participants, irrespective of their completion of treatment, to maximize available study data. With careful planning and involvement of stakeholders in the development of study protocols, a number of trials have demonstrated successful and timely recruitment and retention of pediatric chronic pain participants and their families.^48,63,70^

### 3.4. Technology-based interventions

One way to reduce burden is to intervene with children with chronic pain in more naturalistic settings such as in the home through the use of digital health interventions (ie, delivered through smartphones, websites, text messaging) and to intervene in the school setting. With the almost ubiquitous availability of smartphone and computer technologies, options for delivery of pain treatments in clinical trials have expanded to include web sites, smartphone applications, and videoconferencing.^61^ For children, these forms of remote treatment delivery help to address the barriers that exist for children to participate in chronic pain intervention studies due to the geographical distance that prevents many children from attending study-related clinic visits. An emerging evidence base now exists for internet-delivered psychological interventions for chronic pain in pediatric populations,^63^ with individuals showing improvements in managing pain and disability. However, there are several specific issues with remote treatment delivery in children and adolescents that are important to consider in designing and conducting clinical trials with such treatments. Although the use of technology may appear to be an attractive option, the specific uptake and adherence to technology-based interventions is quite variable between studies. Several strategies have been recommended to address these issues, including use of user-centered and participatory design (incorporating the end user's perspective) and conducting usability and pilot studies to understand issues with intervention delivery and trial design before conducting larger clinical trials.^89^ Technical difficulties with technology and access for families with greater socioeconomic stress are also important considerations. For trials specifically, the missing data that may result from technical problems could lead to loss of statistical power and potential bias in treatment group comparisons. In addition to establishing efficacy, it is equally important to design trials that also establish the likely reach and uptake of digital health interventions through testing implementation, dissemination, and sustainability strategies.

### 3.5. School-based interventions

A potential setting for recruitment and delivery of treatment unique to pediatrics is the school setting. School is the daily “work” of children and represents an important environment for potentially delivering pain prevention and treatment. Schools may be asked to participate in psychological treatment studies (eg, by sending school attendance records or allowing the child to implement practice of behavioral skills during school hours). Moreover, there are successful examples of recruitment into clinical trials for pediatric chronic pain from schools, as well as delivery of treatment at school. For example, Larsson and Carlsson^51^ evaluated the efficacy of a school-based, nurse-administered relaxation training intervention for children with chronic tension-type headache. Developing partnerships that facilitate the conduct of clinical trials in the school setting may help to reduce burden and facilitate recruitment into pediatric chronic pain trials.

### 3.6. Choice of control group and placebo effects

General considerations of the trial architecture are important for enhancing rigor and providing interpretable results. In most situations, a concurrent control group is needed. Although the use of a placebo or attention control group is recommended, many pediatric trials have historically used designs that include no treatment or wait-list control groups. This is clearly a design flaw that can overestimate the effectiveness of a treatment in the absence of some type of credible control group and undermines the interpretation of data. It is recommended that trials use a control group that controls for effects of time, attention, and nonspecific factors related to experimenter–patient interaction that may influence outcomes. For example, in integrative (nonpharmacological) studies, provision of education^48,71^ and unsupervised or standard physical therapy exercise^23,28^ have been used as credible attention control conditions in pediatric chronic pain trials; in pharmacological studies, placebo^77^ or another active treatment^70^ have been most commonly used.

Another issue related to trial design in pediatric chronic pain populations is consideration of placebo effects. There is increasing recognition in pain research that placebo effects can exert a notable influence on trial outcomes in children, adolescents, and adults. This is potentially even more important in pediatric trials, given that children may have stronger placebo responses than adults. A meta-analysis of studies in pediatric migraine demonstrated prominent placebo effects in all trials, with pain relief at 2 hours ranging from 53% to 57.5%.^82^ Another meta-analysis of pharmacologic trials of pediatric headaches showed a decrease in the incidence of headaches from 5.6 to 2.9 per month in placebo groups.^22^ An example of strong placebo effects in the management of pediatric pain was recently highlighted in a large, multisite, rigorously performed randomized clinical trial of headache prevention medications vs placebo in children.^70^ Each group (amitriptyline, topiramate, and placebo) showed strong improvement with ∼65% of patients achieving a clinically meaningful reduction of headaches by ≥50% at the end of 24 weeks of treatment and there were no significant differences between groups. Similarly, in the only double-blinded RCT in children with functional gastrointestinal disorders, both amitriptyline and placebo were associated with excellent therapeutic response with over 50% of the placebo group reporting improvement.^77^ Further work is clearly needed to understand mechanisms of placebo effects in pediatric chronic pain interventions.

### 3.7. Use of innovative clinical trial designs

Innovative clinical trial designs may be particularly important to use in pediatric chronic pain to address issues related to high patient heterogeneity in symptoms and in response to treatment, to address implementation and dissemination strategies for these populations, and as a way to maximize research questions that can be addressed within one sample. As one example, the multiphase optimization strategy framework, adapted from engineering and pioneered by Collins et al.,^13^ is gaining attention in pediatric clinical trials. This is an approach that develops interventions by systematically evaluating potential treatment components to ensure that the intervention is optimized to be maximally efficient and effective. Because no treatment works for all individuals, the sequential multiple assignment randomized trial (SMART) can be used to derive decision rules (ie, algorithms) that specify how alternative interventions or intervention components should be applied optimally to meet the specific and changing needs of individuals.^14^ One advantage for its use in pediatrics is that a sequence of treatments can be studied such as whether medication can be delayed, which is often more acceptable for parents for trial participation, and can provide more information in an efficient manner within the same sample. SMART trials have been used to study sequencing in combined medication and behavioral trials in childhood anxiety and depression^1^ and attention-deficit hyperactivity disorder.^68^ An additional strategy is use of enriched enrollment designs,^58^ which have recently been described in pain clinical trials. A variation of SMART is with Enrichment (SMARTer), which recruits and randomizes additional patients to the second-stage treatments without requiring randomization of the first-stage treatments. This can reduce the sample size of the initial stage and the overall sample size needed for a SMART design.^54^ Last, innovative hybrid effectiveness-implementation trial designs are emerging in other areas of study^15^ and offer an opportunity to combine work on evaluating implementation (eg, testing of an implementation strategy) in the context of gathering information on the clinical intervention's impact on relevant outcomes.

Although these trial designs have not yet been applied to pediatric chronic pain, they are very relevant for addressing the challenge of intervening with children with a wide range of symptom severity and disability (eg, abdominal pain or headaches that are not as yet very disabling and may respond well to minimal interventions and higher levels of care depending on response to lower levels of intervention or level of symptom severity). Moreover, such intervention designs may help to maximize the research questions that can be asked in one sample, which is important, given the more limited samples available for many pediatric pain conditions. To develop prevention and early intervention approaches where treatment is offered as early as possible in the course of chronic pain, innovative designs will be needed. This may be critical to making progress in the prevention of intractable chronic pain into adulthood.

## 4. Developmental concerns in outcome measurement and treatment delivery

Several issues should be considered in outcome measurement in children and adolescents with chronic pain including the children's developmental level, choice of primary and secondary outcomes from core recommended domains, psychometrics of available measures, administration format of measures, choice of informant, and clinical significance of changes in outcome measures. Moreover, developmental issues are important in design and delivery of treatments across the pediatric age span, which covers a broad period of rapid physical, cognitive, and psychological changes.

### 4.1. Developmental level

For chronic pain conditions, most trials have focused on children aged 8 years and above because (1) that is the minimum age at which chronic pain conditions are often first identified, and (2) most children are able to read, understand, and complete outcome measures.^12^ It should, however, be recognized that even within a pediatric age range of 8 to 18 years, there is large variability in intellectual, social, and emotional development that can impact both assessment of outcomes and the implementation of treatments (whether medication, device, psychological, or other type).

### 4.2. Choice of primary and secondary outcomes

Regarding assessment, recommendations for outcome measures have been made by experts convening at a consensus conference, the Pediatric Initiative on Methods, Measurement, and Pain Assessment in Clinical Trials (Ped-IMMPACT). This group identified key outcome domains that are important to assess in clinical trials with children and adolescents who have chronic or recurrent pain.^57^ Eight domains were recommended: pain intensity, physical functioning, symptoms and adverse events, global satisfaction with treatment, emotional functioning, role functioning, sleep, and economic factors. When available, the group recommended use of validated instruments in each of these domains. Since the publication of the Ped-IMMPACT recommendations, there has been an increase in reporting of mood and disability outcomes in trials of children with chronic pain.^28^ We encourage investigators to use these outcome domains as a guide, and to use consistent measures in clinical trials. Furthermore, it is relevant to highlight that these recommendations are within a larger widespread movement of recommendations to use patient-reported outcome data in clinical trials.

### 4.3. Psychometrics of available measures

There are a number of limitations with available pediatric outcome measures. Psychometric data on these measures are often incomplete and limited. Particularly notable are the large gaps in available psychometrics in most commonly used pediatric pain outcome measures for test–retest reliability and sensitivity to change. As one example, Fisher et al.^27^ found that despite the availability, widespread use, and relevance of measures of pain-related anxiety for children and adolescents, very few had psychometric data available beyond basic internal consistency reliability statistics. Moreover, few measures have interpretable cutpoints to allow for interpretation of clinical significance of improvements. Use of 30% or 50% change in pain intensity to define clinically significant change has not been consistently applied in clinical trials in pediatric chronic pain. Often, pain intensity is not the primary outcome but rather disability is, and currently available measures of disability in children generally do not have interpretable change metrics. This has reduced knowledge of clinical improvement in many pediatric chronic pain interventions.

A new set of patient-reported outcome measures is now available with the recent validation of the PROMIS item sets in children, which have undergone a rigorous process of development and validation using modern test theory.^42^ An advantage of PROMIS measures is that they are not disease specific and can be used across different conditions, making comparisons across trials easier in the long run. This is important in pediatric chronic pain for pooling data in meta-analyses across limited RCTs for increased interpretability to develop evidence-based treatment guidelines. At the current time, they are being adopted with caution because the clinical interpretation of these measures and the need to develop clinically meaningful cutpoints is still in process, and the number of pediatric pain populations in which they have been tested in is limited, which might limit their sensitivity. Eventually, it is expected that these short measures may allow for some consistency across trials.

### 4.4. Administration method and choice of informant

Method of administration of study measures and choice of informant are also important considerations. With the increased availability of secure web applications for building and managing online surveys and databases (eg, RedCAP^35^), remote survey administration has become more common in clinical trials. Although this administration method has several strengths including blinded survey administration and reduced participant burden, there are also possible disadvantages such as not being able to easily verify understanding of survey items. Above the age of 8 years, most children can provide a valid self-report of pain, functioning, and psychological symptoms, and we recommend using child self-report as primary. Parent report may provide complementary information but would not typically be recommended as a replacement for child self-report, unless the child cannot provide their own self-report (eg, because of moderate or severe cognitive dysfunction).

### 4.5. Clinical significance

The need for a trial to report results based on statistical significance of findings is clearly important but there has been a call for greater attention to clinical significance for some time. There are mixed opinions on the use of binary endpoints (ie, whether or not the patient achieved clinical improvement based on a cutpoint or not) in clinical trials. Although for some conditions, such as headache and irritable bowel syndrome, there is consensus around what is a meaningful reduction (50% improvement) and on which particular outcome measures, there is not yet consensus for pediatric chronic pain more broadly.

As mentioned, this raises particular challenges for planning a pediatric trial because (1) determination of clinically meaningful change is still in its early phases for pediatric pain measures, and (2) binary endpoints typically require larger sample sizes than do continuous measures for detecting treatment effects, which raises the issue of cost and complexity of multisite studies. Of course, primary endpoints for pain outcomes should map onto the specific standards that impact clinical care (eg, number of headache days in migraine research,^83^ time to a pain flare in arthritis, etc.) as well as be informed by the Food and Drug Administration, and other regulatory standards typical for trials in a specific area. Funding agencies are also moving toward requirements that clinical trials must have adequate power to detect a minimal clinically meaningful difference; stakeholder feedback is needed to set such standards for pediatric chronic pain conditions to apply to clinical trials.

### 4.6. Developmental issues in treatment design and implementation

As mentioned above, developmental levels within the pediatric age range vary considerably and a one-size-fits-all approach to how treatments are designed and implemented cannot be expected to be successful. Designing treatments for different age groups, particularly behavioral interventions, must take into account different attentional and cognitive levels, independence in implementing treatment recommendations, and the relevance to age-appropriate interests and needs. Palermo et al.^65^ identified developmental areas across infancy/toddlerhood, middle childhood, and adolescence that may be important in pain assessment and management. In particular, issues of how best to involve parents and caregivers in treatment of the adolescent present challenges for supporting age-appropriate autonomy and independence while also providing oversight for potential adherence difficulties. Intervention content must also be engaging to the targeted age group, which can be enhanced by obtaining participation of stakeholders in reviewing intervention designs ahead of trials. Moreover, given the smaller available samples for many pediatric chronic pain conditions, there is a tendency for investigators to want to use wide age ranges (eg, 8 to 17 years) to maximize enrollment, which unfortunately runs the risk of inappropriate intervention design and delivery at each end of the range. In general, age effects need to be considered in statistical models, and when possible, study designs should use age stratification, and consider narrow, developmentally defined age groups in trials.

## 5. Ethical considerations

Ethical considerations of clinical trials in children with chronic pain include both general issues pertaining to inclusion of vulnerable populations as well as specific issues related to the conduct of the trial. The obligation to conduct clinical trials to improve children's health and protect them from the risk of using untested interventions must be balanced against protecting children against unknown risks and harms from participation in a clinical trial. Ethical guiding principles in trials for children and adolescents are the same as for adults with chronic pain, and include adhering to the values of respect, patient autonomy and beneficence, nonmaleficence, and justice.^35^ There are additional ethical considerations in pediatric trials, however, because children lack the capacity to understand the risks involved in trials and depend on adults to make decisions for them. There are also additional protections in place for children to guard against potential harms.

### 5.1. Informed consent

Informed consent for participation in pediatric trials is more complex than for adult studies because consent is by proxy from the parent or guardian, who have a duty to protect the child's welfare. In the United States, once children reach 18 years, they are legally able to provide their own consent for study participation; for studies in which participation crosses this age threshold (eg, youth turn 18 years during the study), their reconsent must be obtained at that time point. Regulatory requirements for research involving children include both obtaining and documenting the assent of children (who are cognitively able to provide it) and the consent of the child's guardian. Children's autonomy should be respected and every effort should be made to respect their decision on study participation. There has been some work on developing strategies to aid parents in the decision-making process for trial participation such as improving the readability of the consent document and using innovative methods such as video explanations of study protocols.^36^ One trial in pediatric SCD instituted a peer patient navigator to reduce barriers to study participation and facilitate understanding of the clinical trial,^17^ which may be especially useful for enhancing recruitment in minority populations. Increasingly, the importance of engaging children and families in the recruitment, consent, and design of trials has been recognized.^6^

In addition, the risks and burdens of trial participation must be minimized for children. For example, to protect children from unnecessary testing, the volume of blood sampling generally allowed in pediatric trials is lower than in adults, and it is recommended to obtain samples during routine clinical care whenever possible. Moreover, parents and youth consistently raise study demands as barriers to participation in clinical trials,^4,67^ and thus, strategies to minimize burden are critical in pediatric trials. The majority of parents (91.5%) accept the idea of using placebos in pediatric trials and would prefer enrolling their child in a trial using placebo than in a trial testing the new drug against an already existing drug with possible side effects.^24^

### 5.2. Trial registration and publication

It is an ethical obligation in research for investigators to uphold transparency in the conduct of their trials and to report results through trial registration and publication. All clinical trials should be registered before patient enrollment although an alarmingly high number do not adhere.^34^ Trial discontinuation and nonpublication represent potential waste in resources and have been documented to be a significant problem in pediatric trials, with almost 30% of completed trials not published.^69^ Although not unique to pediatric trials, another important problem identified in pain and anesthesiology clinical trials is accurate, a priori identification of primary and secondary outcome measures.^30,43^ Reviewers and editors serve an important role in identifying issues such as these that compromise interpretability of clinical trials. Given the overall more limited number of pediatric clinical trials, it is particularly important to encourage transparency of trial conduct and reporting of results in investigations in pediatric chronic pain. Researchers seeking to conduct clinical trials of chronic pain interventions in children must consider relevant ethics in the design, implementation, and dissemination of their trials.

## 6. Recommendations and conclusions

As we have highlighted, important considerations exist in designing and conducting clinical trials of chronic pain interventions in children and adolescents, including issues pertaining to sample characterization, treatment administration, developmental considerations in outcome measurement and treatment, and in the ethics of conducting chronic pain trials in children and adolescents. Table 1 summarizes key issues and provides recommendations for addressing them in clinical trials of chronic pain interventions in children and adolescents. Clearly, this is an evolving field and we hope that this article stimulates further discussion of best practices in the design and conduct of clinical trials for children and adolescents with chronic pain.

**Table 1:**
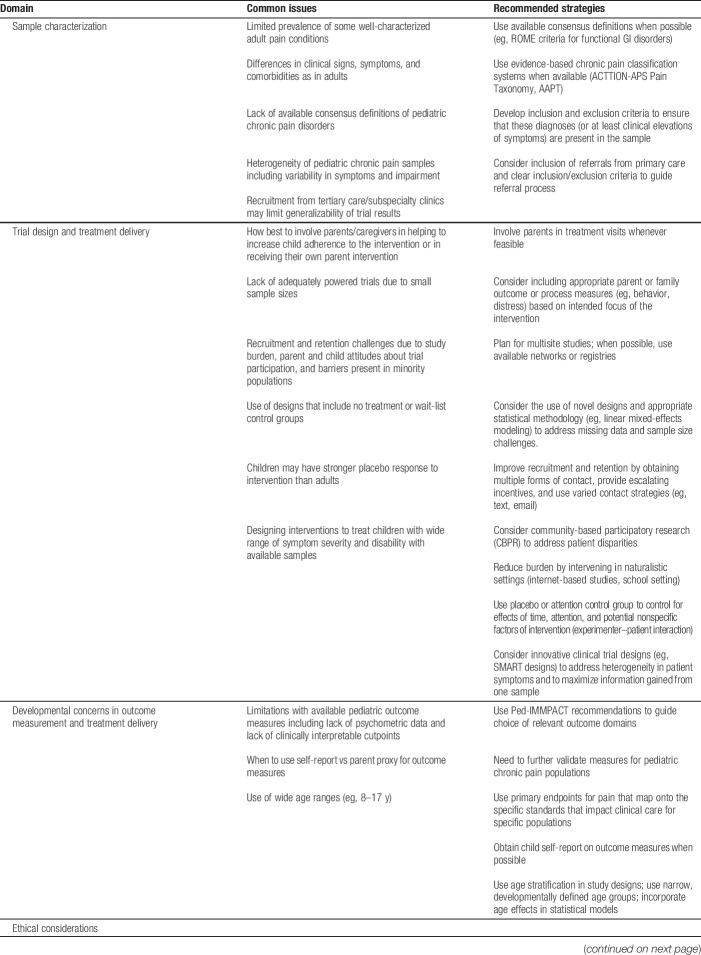

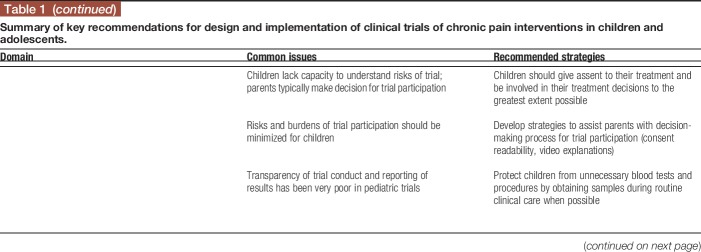
Summary of key recommendations for design and implementation of clinical trials of chronic pain interventions in children and adolescents.

It is important to also emphasize the obligation of quality reporting of clinical trials of chronic pain interventions in children and adolescents. As mentioned, issues of adequate trial registration, nonpublication, and switching of primary outcomes in the analysis phase are all important issues. Moreover, many published trials in children and adolescents have been classified as low quality due to high or unclear risk of bias in other design elements (eg, use of wait-list control conditions). This is in part due to methodological limitations of trial designs but also in part due to lack of proper attention to reporting standards for randomized (CONSORT) and nonrandomized trials (TREND). Greater attention to trial quality and reporting conventions will greatly enhance the literature and confidence in the evidence base for the best treatments for pediatric chronic pain. There are exemplary pediatric chronic pain trials with low risk of bias^48,62,70^ that can serve as models for design and reporting standards.

## Disclosures

The authors have no conflict of interest to declare.

T.M. Palermo is supported in part by Grant no. K24HD060068 from the Eunice Kennedy Shriver National Institute of Child Health and Human Development (NICHD).
